# Changes in Diet Quality from Pregnancy to 6 Years Postpregnancy and Associations with Cardiometabolic Risk Markers

**DOI:** 10.3390/nu15081870

**Published:** 2023-04-13

**Authors:** Jun S. Lai, Marjorelee T. Colega, Keith M. Godfrey, Kok Hian Tan, Fabian Yap, Yap Seng Chong, Yung Seng Lee, Johan G. Eriksson, Shiao-Yng Chan, Mary F. F. Chong

**Affiliations:** 1 Singapore Institute for Clinical Sciences, Agency for Science Technology and Research, Singapore 117609, Singapore; marjorelee_colega@sics.a-star.edu.sg (M.T.C.); obgcys@nus.edu.sg (Y.S.C.); obgjge@nus.edu.sg (J.G.E.); obgchan@nus.edu.sg (S.-Y.C.); mary_chong@nus.edu.sg (M.F.F.C.); 2 MRC Lifecourse Epidemiology Centre & NIHR Southampton Biomedical Research Centre, University of Southampton & University Hospital Southampton NHS Foundation Trust, Southampton SO16 6YD, UK; kmg@mrc.soton.ac.uk; 3 Department of Maternal Fetal Medicine, KK Women’s and Children’s Hospital, Singapore 229899, Singapore; tan.kok.hian@singhealth.com.sg; 4 Duke-NUS Medical School, Singapore 169857, Singapore; fabian.yap.k.p@singhealth.com.sg; 5 Department of Paediatric Endocrinology, KK Women’s and Children’s Hospital, Singapore 229899, Singapore; 6 Department of Obstetrics & Gynaecology and Human Potential Translational Research Programme, Yong Loo Lin School of Medicine, National University of Singapore and National University Health System, Singapore 119228, Singapore; 7 Department of Paediatrics, Yong Loo Lin School of Medicine, National University of Singapore and National University Health System, Singapore 119228, Singapore; paeleeys@nus.edu.sg; 8 Finland and Folkhälsan Research Center, University of Helsinki, Helsinki 00014, Finland; 9 Saw Swee Hock School of Public Health, National University of Singapore and National University Health System, Singapore 117549, Singapore

**Keywords:** diet quality, pregnancy, postpregnancy, adiposity, cardiometabolic

## Abstract

Adopting a healthy diet during and after pregnancy is important for women’s cardiometabolic health. We related changes in diet quality from pregnancy to 6 years postpregnancy to cardiometabolic markers 8 years postpregnancy. In 652 women from the GUSTO cohort, we assessed dietary intakes at 26–28 weeks’ gestation and 6 years postpregnancy using 24 h recall and a food frequency questionnaire, respectively; diet quality was scored using a modified Healthy Eating Index for Singaporean women. Diet quality quartiles were derived; stable, large/small improvement/decline in diet quality as no change, >1 or 1 quartile increase/decrease. Fasting triglyceride (TG), total-, high- and low-density-lipoprotein cholesterol (TC, HDL- and LDL-C), glucose and insulin were measured 8 years postpregnancy; homeostatic model assessment for insulin resistance (HOMA-IR) and TG: HDL-C ratio were derived. Linear regressions examined changes in diet quality quartiles and cardiometabolic markers. Compared to a stable diet quality, a large improvement was associated with lower postpregnancy TG [−0.17 (−0.32, −0.01) mmol/L], TG: HDL-C ratio [−0.21 (−0.35, −0.07) mmol/L], and HOMA-IR [−0.47 (−0.90, −0.03)]; a large decline was associated with higher postpregnancy TC and LDL-C [0.25 (0.02, 0.49); 0.20 (0.004, 0.40) mmol/L]. Improving or preventing a decline in diet quality postpregnancy may improve lipid profile and insulin resistance.

## 1. Introduction

There is evidence that adopting a healthy diet during pregnancy is associated with better pregnancy outcomes such as lower risks of gestational diabetes [1] and preterm birth [2], as well as better offspring metabolic and cognitive health [3,4]. Having a healthy diet after pregnancy is also important to ensure optimal maternal health in the long term. A woman’s diet after pregnancy can influence postpartum weight retention [5], which contributes to the risk of cardiometabolic diseases later in life [6,7].

Evaluating changes in diet during transitional life stages such as from pregnancy to postpregnancy could identify a window of opportunity for interventions to reduce disease risk. A recent systematic review examining changes in diet from pregnancy to postpregnancy found a general decline in healthy dietary behaviors/patterns postpregnancy [8], possibly due to the demands associated with caring for a child [9]. During the transition from pregnancy to postpregnancy, women significantly decreased their fruit and vegetable intakes, decreased diet quality or adherence to a healthy dietary pattern, whilst significantly increasing discretionary food intakes [8]. Similarly, using data from a longitudinal mother–offspring cohort of multiethnic Asian women, we previously observed that approximately 30% of mothers decreased adherence to a dietary pattern characterized by higher intakes of fruit, vegetables, plant proteins and whole grains, as well as the emergence of an “unhealthy” dietary pattern postpregnancy which was not observed during pregnancy, suggesting poorer dietary intake postpregnancy [10].

Whilst the aforementioned studies assessed changes in diet from pregnancy to postpregnancy, they only described the correlates or determinants of these changes [8,10], and did not relate dietary changes from pregnancy to postpregnancy with cardiometabolic risk markers (CMRMs). Evaluating the impact of dietary changes on CMRMs is meaningful to determine the associated changes in risk markers when individuals make changes to their diet. Understanding how improvements in diet quality influence subsequent CMRMs allows us to mimic an intervention study where individuals make real-life changes to their diet quality.

It can be expected that long-term maintenance of a high-quality diet from pregnancy to postpregnancy associate with lower cardiometabolic risk due to mounting evidence in nonpregnant populations showing a high adherence to healthful dietary patterns to associate with lower cardiovascular disease (CVD) risk [11]. However, to consistently maintain a high-quality diet is challenging, and as aforementioned, women tended to decrease their diet quality after pregnancy. It is unclear whether a deterioration in diet quality will make a difference in women’s cardiometabolic disease risk.

The present study aimed to examine the associations between changes in diet quality from pregnancy to 6 years postpregnancy and CMRMs (adiposity, lipid profile, glycaemia, insulin resistance, and blood pressure) at 6–8 years postpregnancy in a longitudinal cohort of multiethnic Asian women.

## 2. Materials and Methods

### 2.1. Study Sample

We used data from the Growing Up in Singapore Towards healthy Outcomes (GUSTO) study—a mother–offspring cohort in Singapore [12]. GUSTO recruited pregnant women (≥18 years) in their first trimester (<14 weeks) from the National University Hospital and KK Women’s and Children’s Hospital—major public maternity units in Singapore during June 2009–September 2010. Only Chinese, Malay or Indian women of Singapore citizenship or permanent residency, with homogenous parental ethnic background, were eligible to participate; women receiving chemotherapy or psychotropic drugs or who had type I diabetes mellitus were ineligible. Further details on the GUSTO study have been published [12]. All procedures of the GUSTO study were approved by the Institutional Review Board of the two maternity units, and in accordance with the Declaration of Helsinki. Written informed consent was obtained from all participants at study recruitment.

**Figure 1:**
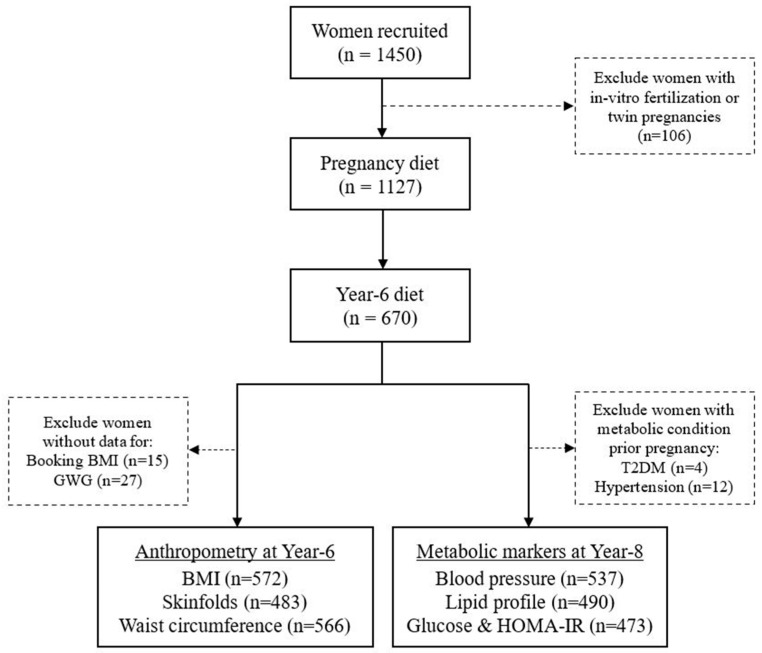
Participant flowchart. BMI, body mass index; GWG, gestational weight gain; HOMA-IR, homeostasis model assessment of insulin resistance; T2DM, type-2 diabetes mellitus.

GUSTO recruited 1450 women initially, but the present analysis excluded women who conceived via in vitro fertilization or had twin pregnancies (*n* = 106); only 652 women had complete data for dietary intake during pregnancy and at 6 years postpregnancy, as well as data for at least one measurement of CMRMs (adiposity, lipid profile, glycaemia, insulin resistance, and blood pressure) at 6–8 years postpregnancy. For analyses with adiposity, women were further excluded if they did not have data for booking BMI and GWG, whereas for analyses with glycaemia and HOMA-IR or blood pressure, women were excluded if they self-reported having pre-existing T2DM or hypertension at recruitment, respectively (Figure 1).

### 2.2. Dietary Intakes during Pregnancy and at 6 Years Postpregnancy

Women’s dietary intake during pregnancy was assessed at 26–28 weeks’ gestation with a 24 h recall. The 24 h recall was administered by trained research staff using the 5-stage, multiple-pass interviewing technique [13]. Visual aids in terms of household utensils and portion-size pictures were provided to assist in estimation of amounts consumed. Further details of the 24 h recall procedures and analyses in the GUSTO study have been previously published [14,15].

Dietary intake of these women was reassessed at 6 years postpregnancy with a 133-item, semi-quantitative food frequency questionnaire (FFQ), which was administered by trained research staff [10]. Further details and validation of the FFQ have been published [10,16]. In brief, women were asked to indicate their frequency of consuming each FFQ item in the past 1 month in an open-ended format (“never”, “number of times per month”, “number of times per week” or “number of times per day”), and the average amount consumed. Images of household utensils and portion sizes were provided.

Nutrient analysis of the 24 h recalls and FFQs was performed using the Dietplan software (Forestfield Software Ltd., West Sussex, UK) based on a local food composition database.

### 2.3. Diet Quality during Pregnancy and at 6 Years Postpregnancy

Diet quality during pregnancy was ascertained using the Healthy Eating Index for pregnant women in Singapore (HEI-SGP) [17]. The HEI-SGP was developed with reference to the Healthy Eating Index [18] and the Alternate Healthy Eating Index for Pregnancy [19], modified according to the Singapore dietary guidelines for pregnant women [20]. The original HEI-SGP has 11 components and a maximum possible score of 90, with a higher score indicating better diet quality. Each component was scored based on nutrient density per 1000 kcal, with the exception of total fat and saturated fat. Total fruits, whole fruits, total vegetables, and dark green leafy and orange vegetables were scored 5 if recommendations were met, 0 for no consumption, and proportionately for intermediate intakes. Total rice and alternatives, whole grains, dairy and total protein foods were scored 10 if recommendations were met, 0 for no consumption, and proportionately for intermediate intakes. Total fat and saturated fat were scored 10 if recommendations were met (<30% and <10% of energy intake, respectively), scored 0 if >40% and >20% of energy intake, respectively, and proportionately for intermediate intakes (30–40% and 10–20% of energy intake, respectively). The consumption of antenatal supplements containing iron, folate, and calcium was scored 10 if the supplements contained all three micronutrients, 5 if containing one or two of these stated micronutrients, and 0 if not consumed or the supplements did not contain these micronutrients. Details of the HEI-SGP were previously published [17].

For the ascertainment of diet quality at 6 years postpregnancy, we modified the HEI-SGP according to local recommendations for nonpregnant women [21]. The main changes were in the recommended intakes due to differences in dietary requirements between pregnant and nonpregnant women. Additionally, the dietary supplements component was removed from calculation of the modified HEI-SGP scores at 6 years postpregnancy as there are no local recommendations for dietary supplements intake for nonpregnant women [21]. The assignment of scores and foods categorized under each specific component was similar, with differences or similarities in scoring between pregnancy and 6 years postpregnancy shown in Supplementary Table S1.

To ensure comparability of diet quality scores between the time points for subsequent analyses, the antenatal supplements component was removed from the calculation of scores at pregnancy, resulting in maximum scores of 80 for both time points instead of the original maximum score of 90.

### 2.4. Adiposity at 6 Years Postpregnancy

At 6 years postpregnancy, women’s weight was measured using an electronic weighing scale to the nearest 0.1 kg, and height was measured with a stadiometer (SECA Corp, Hamburg, Germany) to the nearest 0.1 cm by trained research staff. Body mass index (BMI) was calculated as weight (in kilograms) divided by height (in meters and squared). Waist circumference (WC) was measured at the uppermost lateral border of the ilium to the nearest 0.1 cm using nonstretchable measuring tape. Skinfold thicknesses were measured to the nearest 0.2 mm at four sites (biceps, triceps, subscapular and suprailiac) using a Holtain skinfold caliper following standard procedures [22]. Sum of skinfold thicknesses (SST) at all four sites were derived. For reliability, weight, height, and waist circumference were taken in duplicate, while skinfold measurements were taken in triplicate, and respective measurements were averaged.

### 2.5. Cardiometabolic Risk Markers at 8 Years Postpregnancy

At 8 years postpregnancy, overnight fasting plasma triglycerides (TG), total cholesterol (TC), low-density-lipoprotein cholesterol (LDL-C), high-density-lipoprotein cholesterol (HDL-C), glucose and insulin were measured using standard colorimetric or enzymatic methods in a clinically accredited laboratory. Homeostasis model assessment of insulin resistance (HOMA-IR) was calculated as (fasting insulin [mU/L] × fasting glucose [mmol/L])/22.5. Ratios of TC to HDL-C (TC: HDL-C) and of TG to HDL-C (TG: HDL-C) were derived.

Peripheral systolic blood pressure (SBP) and diastolic blood pressure (DBP) were measured in triplicate (Dinamap CARESCAPE V100, GE Healthcare, Milwaukee, WI, USA) from the upper right arm by trained research staff following standardized protocols, and the measurements were averaged.

### 2.6. Derivation of Framingham Risk Score

The Framingham risk score (FRS) was used to estimate women’s risk of CVD over 10 years, with higher scores indicating higher CVD risk [23]. The scores were calculated based on age, sex, elevated TC levels, low levels of HDL-C, cigarette smoking, and SBP or hypertension diagnosis [23] assessed at 8 years postpregnancy. Information on current cigarette smoking and being diagnosed with hypertension was self-reported via questionnaires administered by trained research staff. We modified the FRS, which was originally scored according to the Framingham-based NCEP ATP III 10-Year Risk Score Tables [24], to account for local clinical practice guidelines for lipids and blood pressure management. Detailed scoring of the locally modified FRS [25] as well as local clinical practice guidelines have been published [26,27].

### 2.7. Covariates

At study recruitment, information on women’s age, ethnicity, highest education attained, monthly household income, type-2 diabetes mellitus (T2DM) and high blood pressure prior to pregnancy was collected via self-report. Parity at recruitment was retrieved from hospital delivery records. Women’s BMI at first antenatal appointment (booking BMI) was determined based on weight measured at first antenatal appointment (in the first trimester), and height measured at the 26–28 weeks’ gestation study visit. Inadequate, adequate and excessive gestational weight gain (GWG) were according to the cut-offs set by the Institute of Medicine recommended rate of weight gain (kg/week) in the second and third trimesters [28] based on booking BMI category. Methods deriving the rate of GWG have been detailed elsewhere [29]. At 26–28 weeks’ gestation, self-reported physical activity in the past 7 days was assessed with the International Physical Activity Questionnaire (IPAQ) (31). Duration and frequency of physical activity were used to derive metabolic equivalent minutes per week (MET-min/week) and categorized as follows: <600, 600–3000, and >3000 MET-min/week for insufficiently, sufficiently or highly active, as detailed previously [30]. Women underwent a 2 h 75 g oral glucose tolerance testing at 26–28 weeks’ gestation to determine the presence of gestational diabetes mellitus (GDM) according to the 1999 WHO diagnostic criteria [31]. Information on hypertensive disorders of pregnancy (pre-eclampsia and pregnancy-induced hypertension) was obtained from hospital case notes. Education and household income were reassessed at 5 years postpregnancy, and physical activity was reassessed at 6 years postpregnancy. Updated parity information at 8 years postpregnancy was derived by summing the number of births after the GUSTO birth, GUSTO birth, and parity at recruitment, as women’s cardiometabolic risk increases with increasing parity [32]. Weight changes from pregnancy to 8 years postpregnancy were calculated as the difference between the measured weight at first antenatal appointment and measured weight at 8 years postpregnancy.

### 2.8. Statistical Analysis

#### 2.8.1. Primary Analysis

Quartiles of diet quality scores at pregnancy and postpregnancy were derived separately. A change in diet quality was computed as the difference in quartiles of scores at pregnancy and postpregnancy. The women were categorized into 5 groups of change in diet quality as follows: stable (no change in quartile), large decrease (>1 quartile decrease), small decrease (1 quartile decrease), small increase (1 quartile increase), and large increase (>1 quartile increase).

Participant characteristics according to groups of change in diet quality were compared using one-way ANOVA for continuous variables or chi-squared tests for categorical variables.

Linear regressions were performed to examine associations between changes in diet quality (5 groups: stable, large/small decrease, and large/small increase) and adiposity and CMRMs at 6–8 years postpregnancy. Models were adjusted for age at recruitment, ethnicity, education and household income at recruitment and their changes at 5 years postpregnancy, updated parity at 8 years postpregnancy, physical activity at midpregnancy and change at 6 years postpregnancy, booking BMI, and quartiles of pregnancy diet quality scores. Models with adiposity outcomes were additionally adjusted for GWG category, whilst models with CMRMs were additionally adjusted for weight changes from pregnancy to 8 years postpregnancy to determine if changes in markers were a result of changes in weight, as well as (1) GDM for analysis of glycaemia and HOMA-IR outcomes, and (2) hypertensive disorders of pregnancy for analysis of blood pressure outcome.

#### 2.8.2. Secondary Analysis

In addition to examining the influence of dietary changes, we examined the influence of diet quality at specific time periods, i.e., pregnancy or postpregnancy on CMRMs by performing additional analyses to separately associate diet quality at pregnancy and at 6 years postpregnancy with adiposity and CMRMs postpregnancy, with mutual adjustment for diet quality at the other time point. The models associating diet quality at pregnancy with outcomes were adjusted for diet quality at 6 years postpregnancy, ethnicity, booking BMI, and midpregnancy physical activity; age, education, household income and parity at recruitment; and GWG category (for adiposity), or weight changes at 8 years postpregnancy and GDM (for glycaemia and HOMA-IR), or weight changes at 8 years postpregnancy and hypertensive disorders of pregnancy (for blood pressure). The models associating diet quality at 6 years postpregnancy with outcomes were adjusted for diet quality at pregnancy, age at recruitment, ethnicity, and booking BMI; education and household income at 5 years postpregnancy; parity and weight changes at 8 years postpregnancy; and GWG category (for adiposity), GDM (for glycaemia and HOMA-IR) or hypertensive disorders of pregnancy (for blood pressure).

We also tested effect modification by parity at recruitment (0 and ≥1) by adding interaction terms (parity × groups of change in diet quality) in the multivariable regression models, and subsequently performed stratified analysis for any statistically significant interactions.

To investigate how each of the 10 HEI-SGP components contributed to the association between a change in diet quality and adiposity or CMRMs, we successively excluded each component at both pregnancy and postpregnancy and compared the attenuation in effect estimates.

Multiple imputation with chained equations (20 times) were performed for covariates with missing data: education (n = 1), household income (n = 14), parity (n = 48) and physical activity (n = 36) collected in the postpregnancy period. We used Stata version 14 (StataCorp LP, College Station, TX, USA) to perform all analyses, and considered two-sided *p* < 0.05 to be statistically significant.

## 3. Results

### 3.1. Primary Analysis

Of the 652 women with data for at least 1 outcome, 18.3% (n = 119) and 16.4% (n = 107) had a large decrease or increase in diet quality, respectively; 16.7% (n = 109) and 19.6% (n = 128) had a small decrease or increase in diet quality, respectively; and 29% (n = 189) remaining in the same diet quality quartile from pregnancy to 6 years postpregnancy. Comparisons of characteristics across changes in diet quality are shown in Table 1. Women with a decrease (small or large) in diet quality tended to be older and of Chinese ethnicity, whereas women with an increase (small or large) in diet quality tended to be of Malay ethnicity.

When examining the associations with adiposity at 6 years postpregnancy, there were no associations between change in diet quality and the BMI, sum of skinfolds and waist circumference (Table 2).

When examining the associations with CMRMs at 8 years postpregnancy, a “large decrease” in diet quality was associated with a 0.25 mmol/L (95% CI: 0.02, 0.49) higher total cholesterol and 0.20 mmol/L (95% CI: 0.004, 0.40) higher LDL-C (Table 2), compared to a “stable” diet quality. Additionally, a “large increase” in diet quality was associated with 0.17 mmol/L (95% CI: −0.32, −0.01) lower triglycerides and 0.21 (95% CI: −0.35, −0.07) lower TG: HDL-C ratio, as well as 0.47 (95% CI: −0.90, −0.03) lower HOMA-IR, compared to a “stable” diet quality. A “small” decrease/increase in diet quality was not associated with the same CMRM at 8 years postpregnancy. No associations were observed for change in diet quality with HDL-C, TC: HDL-C ratio, fasting glucose, blood pressure, and FRS.

### 3.2. Secondary Analysis

Compared to the lowest quartile of diet quality, being in the highest quartile of diet quality at pregnancy was associated with a lower sum of skinfolds and lower waist circumference at 6 years postpregnancy, as well as a lower FRS at 8 years postpregnancy (Table 3), but no associations were observed between diet quality at 6 years postpregnancy and these risk markers. Additionally, being in the highest quartile of diet quality at either pregnancy or 6 years postpregnancy was associated with lower triglycerides, TC: HDL-C ratio, TG: HDL-C ratio, and HOMA-IR, compared to the lowest quartile of diet quality.

Parity at recruitment significantly modified the associations between changes in diet quality and total cholesterol and LDL-C (P-interaction < 0.05), whereby the associations between a “large decrease” in diet quality and higher total and LDL cholesterol were significant and stronger among women who were parous at the study recruitment stage (Supplementary Table S2).

Successively excluding fruit (total or whole), dairy, and protein food components at both time points attenuated the association between a “large decrease” and higher total cholesterol and LDL cholesterol (Supplementary Figure S1). Successively excluding the dairy component and/or total rice and alternatives and protein food components at both time points attenuated the association between a “large increase” and lower triglycerides and TG: HDL-C ratio (Supplementary Figure S2). Successively excluding the whole grains, dairy, and saturated fat components at both time points attenuated the association between a “large increase” in diet quality and lower HOMA-IR.

## 4. Discussion

In a cohort of multiethnic Asian women, we found that a large improvement in diet quality (assessed using a modified HEI-SGP) from pregnancy to 6 years postpregnancy was associated with lower triglyceride levels and insulin resistance, whereas a large decline in diet quality was associated with higher cholesterol levels. High diet quality during pregnancy was associated with a lower sum of skinfolds and waist circumference at 6 years postpregnancy, as well as a lower predicted 10-year CVD risk at 8 years postpregnancy, but a change in diet quality from pregnancy to 6 years postpregnancy was not associated with these risk markers.

To the best of our knowledge, this is the first study to examine changes in women’s diet quality from pregnancy to postpregnancy with postpregnancy CMRMs. Undeniably, women who maintained a high diet quality from pregnancy to postpregnancy had the lowest levels of risk markers (Supplementary Table S3), but we additionally showed that making improvements to/preventing a decline in diet quality postpregnancy can still confer benefits on lipid profile and insulin resistance. Our results are reminiscent of findings from a longitudinal study of diet quality with metabolic outcomes in adult men and women, whereby an increase in a priori dietary scores (e.g., the Portfolio diet, the Dietary Approaches to Stop Hypertension diet score, or the healthy diet score) was associated with a lowering of several CMRMs [33] (e.g., triglycerides, cholesterol, glucose, HbA1c, and blood pressure) as well as a lower risk of T2DM [34]. These results, when considered together with findings from randomized control trials (e.g., the PREDIMED study [35] and the Lyon Diet Heart study [36]) support improvements in overall diet as an important strategy to improve CMRMs. In our study, we found that a large improvement in diet quality, rather than a small improvement, is required to achieve favorable changes in CMRMs, which can be achieved by changing multiple dietary factors such as consuming greater amounts and varieties of fruit and vegetables, more whole grains, and less fat and saturated fat. Health promotion programs supporting women to make improvements in several dietary aspects postpregnancy are needed to impact women’s long-term cardiometabolic health.

An inherent issue in examining changes in diet quality is that the corresponding changes in CMRMs may be dependent on the initial diet quality. For example, participants with a larger increase in diet quality tended to be those with a poorer diet quality initially. However, our analyses (adjusting for diet quality at pregnancy) showed that those with a large increase in diet quality had lower triglycerides and insulin resistance independent of the initial diet quality, suggesting that improving women’s diet after pregnancy can be beneficial for these risk markers. In addition, this study provides novel data showing a deterioration in diet quality is associated with higher cholesterol independent of the initial diet quality. Concurring with our previous publication [10], in which a third of women were observed to decrease adherence to a “Fruit, vegetables and legumes” dietary pattern (a “healthy” diet), approximately 35% of women in the present study decreased their diet quality postpregnancy, with 18% having a large decrease. This is concerning and signifies the need for more interventions and health promotion efforts to prevent a decline in diet quality during the transition from pregnancy to motherhood.

The associations between changes in diet quality and lipids and insulin resistance did not appear to be explained by weight changes from pregnancy to 8 years postpregnancy. The potential mechanisms explaining the associations may be multifactorial because the modified HEI-SGP includes multiple food components, for example, antioxidants from fruit and vegetables may reduce the oxidative stress contributing to the pathogenesis of cardiometabolic diseases [37], whilst saturated fatty acids are proinflammatory molecules contributing to elevated cholesterols and insulin resistance [38].

We did not find significant associations between changes in diet quality and adiposity at 8 years postpregnancy, possibly because the total energy intake or amounts eaten play more important roles in adiposity [39], whilst the modified HEI-SGP measures diet quality independent of total energy intake and quantity (i.e., each dietary component is standardized for energy intake). Alternatively, it is possible that adiposity reflects the longer-term diet rather than shorter-term dietary changes; as such, diet quality during pregnancy may play a more important role. This is likely because we found that being in the highest quartile of diet quality scores during pregnancy was associated with a lower sum of skinfolds and waist circumference, independent of diet quality at 6 years postpregnancy.

The association between a large decrease or increase in diet quality and FRS at 8 years postpregnancy was in the same direction as the associations with lipids and insulin resistance, but did not reach statistical significance. One possible reason could be that our cohort was generally made up of participants with very low CVD risk (i.e., young participants with a mean age of 39.6 years, few (n = 38) of whom smoked), hence limiting the variation needed to detect significant associations. Another possible reason is that the risk markers shown to have associations with changes in diet quality were not included as components of the FRS. Similar to the findings with adiposity, having a high diet quality during pregnancy may promote favorable cardiometabolic outcomes later in life rather than a high diet quality at 6 years postpregnancy, highlighting the importance of early intervention for a reduction in CVD risk.

Additionally, neither a change in diet quality nor diet quality at pregnancy and 6 years postpregnancy was associated with blood pressure, likely because the modified HEI-SGP did not capture the aspects of diet most closely linked to blood pressure. It is well established that a reduction in sodium or salt intake decreases blood pressure and the incidence of hypertension [40], but this was not included as a component of the modified HEI-SGP.

We noted that fruit, dairy, and protein foods are important contributors to the association between a large decline in diet quality and higher total and LDL cholesterol, as excluding these components attenuated the associations. Indeed, a higher fruit intake has been associated with lower total and LDL cholesterol levels [41]. The beneficial role of dairy and protein foods for total and LDL cholesterol observed in our study may be attributable to higher intakes of low-fat dairy and plant-based protein foods [42,43,44]. This may also explain the contribution of dairy to the associations between a large improvement in diet quality and triglycerides and TG: HDL-C ratio. However, the scoring of the HEI-SGP did not differentiate between the types of dairy and protein foods consumed, which warrants further investigation. Additionally, whole grains and saturated fat appear to be major contributors to the associations between a large improvement in diet quality and HOMA-IR, concurring with previous studies which reported that higher intakes of whole grains and limiting the intake of saturated fat may be beneficial for insulin resistance [45,46].

We found a significant effect modification by parity at recruitment in that the associations between a large decline in diet quality and higher total and LDL cholesterols levels was stronger among women who were parous at study recruitment. Previous research has shown that women with more children reported a lower diet quality [19] as well as multiparity to be a risk factor of cardiometabolic diseases later in life [47], which, when considered together, may have multiplicative effects on cardiometabolic outcomes, as shown in the current study findings.

The strengths of this study include the prospective, longitudinal design with repeated measures of dietary intake. The use of a dietary index with standardized dietary components and scoring criteria allowed the assessment of changes in diet quality as opposed to using data-driven dietary patterns, which are often not reproducible across time points. Furthermore, unlike data-driven dietary patterns, which may not necessarily define the healthiest patterns, our modified HEI-SGP was constructed according to local dietary guidelines on what constitutes a healthy diet, which allows translation into practical recommendations. Several limitations should be noted. The CMRMs were measured at 8 years postpregnancy, with diet quality reassessed at 6 years postpregnancy; there may be changes in diet between 6 years and 8 years postpregnancy which may have changed the outcome(s) of interest, which we did not account for. Different dietary assessment methods were used at the two time points (i.e., 24 h recall during pregnancy and the FFQ at 6 years postpregnancy), which may have affected the comparability of diet quality scores, but we used the change in the quartiles of diet quality scores instead of absolute scores to account for this difference in the assessment method. Furthermore, we showed in our previous study that dietary patterns derived using the two-assessment method can be tracked longitudinally [10]. Diet was reassessed 6 years after pregnancy, which is a long time after pregnancy, and we could not ascertain when the changes were made and whether the timing of this change and how long the change was sustained influenced the associations observed. As with any longitudinal cohorts, many participants were lost to follow-up due to having a busy schedule, inconvenience, and no longer wishing to participate because their children had grown up; although the current analysis was amongst 652 of 1450 women initially recruited, we showed in a previous publication that the participant characteristics were similar between those with dietary data at both time points versus those who did not [10]. The GUSTO study did not intentionally recruit women representative of the general Singaporean population; hence, our findings may not be generalizable to the general Singaporean population nor to populations of differing ethnicities and socioeconomic status.

## 5. Conclusions

Our study found that a large improvement in diet quality was associated with better CMRMs and a large decline in diet quality was associated with worse risk markers. This highlights the need to support women beyond the pregnancy and early postpartum period to improve overall diet quality for better CMRMs, with parous women requiring greater support to improve their diet quality postpregnancy. Pregnancy may be an opportune time to encourage the adoption of high-quality diets to promote favorable long-term adiposity outcomes and lower CVD risk, but this finding will require replication in other studies.

## Figures and Tables

**Table 1:** Characteristics ^1^ of GUSTO women included in the analysis of change in diet quality with cardiometabolic risk markers (n = 652).

	Large Decrease	Small Decrease	Stable	Small Increase	Large Increase	*p* ^2^
	(n = 123)	(n = 107)	(n = 193)	(n = 124)	(n = 105)	
Age at recruitment, year	30.9 ± 5.0	31.9 ± 5.1 *	30.2 ± 4.9	30.9 ± 5.3	29.7 ± 5.3 *	0.021
Ethnicity						<0.001
Chinese	89 (72.4)	68 (63.6)	110 (57.0)	59 (47.6)	43 (41.0)	
Malay	22 (17.9)	21 (19.6)	46 (23.8)	43 (34.7)	39 (37.1)	
Indian	12 (9.8)	18 (16.8)	37 (19.2)	22 (17.7)	23 (21.9)	
Highest Education						0.160
Primary/secondary	36 (29.3)	33 (30.8)	56 (29.2)	42 (33.9)	41 (39.1)	
Postsecondary	40 (32.5)	33 (30.8)	61 (31.8)	48 (38.7)	39 (37.1)	
University	47 (38.2)	41 (38.3)	71 (39.1)	34 (27.4)	25 (23.8)	
Monthly household income, SGD						0.054
<1999	14 (12.2)	15 (15.3)	29 (16.2)	20 (16.9)	20 (20.4)	
2000–5999	64 (55.6)	57 (58.2)	90 (50.3)	76 (64.4)	61 (62.2)	
>6000	37 (32.2)	26 (26.5)	60 (33.5)	22 (18.6)	17 (17.4)	
Booking BMI ^3^, kg/m^2^	23.3 ± 4.1	23.3 ± 4.6	23.4 ± 4.4	24.9 ± 5.9	23.4 ± 4.5	0.053
Parity at recruitment						0.161
0	56 (45.5)	36 (33.6)	87 (45.1)	56 (45.2)	40 (38.1)	
≥1	67 (54.5)	71 (66.4)	106 (54.9)	68 (54.8)	65 (61.9)	
Gestational diabetes	26 (21.9)	22 (21.4)	30 (16.0)	21 (17.7)	11 (10.5)	0.313
Pregnancy hypertensive disorders	6 (4.9)	6 (5.6)	9 (4.7)	8 (6.5)	1 (1.0)	0.273
Gestational weight gain						0.324
Excessive	57 (47.9)	50 (49.5)	98 (53.3)	70 (59.3)	51 (53.1)	
Inadequate	11 (9.2)	13 (14.1)	28 (15.2)	9 (7.6)	12 (12.5)	
Normal	51 (42.9)	36 (36.4)	58 (31.5)	39 (33.1)	33 (34.4)	
Physical activity, MET-min/week						0.398
<600	39 (32.0)	32 (30.2)	64 (33.3)	40 (32.5)	24 (22.9)	
600–3000	57 (46.7)	57 (53.8)	90 (46.9)	65 (52.9)	54 (51.4)	
3000	26 (21.3)	17 (16.0)	38 (19.8)	18 (14.6)	27 (25.7)	

BMI, body mass index; GUSTO, Growing Up in Singapore Towards healthy Outcomes; MET, metabolic equivalent of task; ^1^ Values are mean ± SD or n (%). Characteristics were based on data obtained during study recruitment or 26–28 weeks gestation unless otherwise specified. ^2^ *p*-values are for one-way ANOVA (* mean values in a row with a common symbol differ, *p*< 0.05 based on Bonferroni post hoc analysis) or chi-square tests; ^3^ Based on weight measured at first antenatal appointment in the first trimester and height measured at 26–28 weeks’ gestation.

**Table 2:** Associations between change in diet quality from pregnancy to 6 years postpregnancy and anthropometry and cardiometabolic markers at 6–8 years postpregnancy in women of the GUSTO cohort.

	Large Decrease		Small Decrease		Stable	Small Increase		Large Increase	
	β (95% CI)	*p*	β (95% CI)	*p*		β (95% CI)	*p*	β (95% CI)	*p*
Anthropometry ^1^									
BMI, kg/m^2^	−0.34 (−0.96, 0.29)	0.294	−0.11 (−0.72, 0.49)	0.711	Reference	0.11 (−0.45, 0.68)	0.694	−0.41 (−1.05, 0.24)	0.219
Skinfolds ^2^, mm	−0.46 (−5.45, 4.53)	0.647	1.12 (−3.67, 5.90)	0.647	Reference	3.36 (−1.14, 7.87)	0.143	−0.93 (−6.24, 4.37)	0.730
WC ^3^, cm	−0.11 (−2.08, 1.85)	0.909	0.97 (−0.94, 2.87)	0.318	Reference	0.59 (−1.17, 2.36)	0.510	−1.16 (−3.22, 0.91)	0.271
Lipid profile ^4^									
Total cholesterol, mmol/L	0.25 (0.02, 0.49)	0.032	−0.08 (−0.32, 0.16)	0.520	Reference	0.04 (−0.18, 0.26)	0.731	0.05 (−0.20, 0.29)	0.707
Triglycerides, mmol/L	0.003 (−0.11, 0.12)	0.955	−0.004 (−0.14, 0.13)	0.960	Reference	−0.13 (−0.27, 0.01)	0.067	−0.17 (−0.32, −0.01)	0.038
LDL-C, mmol/L	0.20 (0.004, 0.40)	0.046	−0.07 (−0.27, 0.14)	0.524	Reference	0.05 (−0.13, 0.24)	0.581	0.06 (−0.15, 0.27)	0.578
HDL-C, mmol/L	0.05 (−0.03, 0.13)	0.222	−0.01 (−0.09, 0.06)	0.755	Reference	0.04 (−0.03, 0.11)	0.214	0.06 (−0.02, 0.14)	0.130
TC: HDL-C	0.15 (−0.08, 0.37)	0.204	−0.06 (−0.28, 0.17)	0.624	Reference	−0.13 (−0.34, 0.07)	0.192	−0.17 (−0.40, 0.07)	0.158
TG: HDL-C	−0.01 (−0.15, 0.13)	0.900	0.003 (−0.13, 0.14)	0.960	Reference	−0.16 (−0.29, −0.04)	0.012	−0.21 (−0.35, −0.07)	0.004
Glycemia ^5^									
Fasting glucose, mmol/L	−0.04 (−0.35, 0.27)	0.800	0.01 (−0.30, 0.32)	0.945	Reference	0.06 (−0.22, 0.34)	0.655	−0.09 (−0.26, 0.08)	0.317
HOMA-IR	0.23 (−0.21, 0.66)	0.304	−0.002 (−0.40, 0.40)	0.991	Reference	−0.07 (−0.47, 0.33)	0.728	−0.47 (−0.90, −0.03)	0.035
Blood pressure ^6^									
Systolic, mmHg	−0.55 (−4.23, 3.13)	0.769	2.43 (−1.19, 6.04)	0.188	Reference	−1.27 (−4.57, 2.03)	0.450	−0.17 (−4.03, 3.68)	0.931
Diastolic, mmHg	−1.23 (−3.89, 1.44)	0.366	0.53 (−2.09, 3.16)	0.690	Reference	−0.40 (−3.20, 2.40)	0.779	−1.85 (−4.25, 0.46)	0.070

BMI, body mass index; GUSTO, Growing Up in Singapore Towards healthy Outcomes; HDL-C, high-density-lipoprotein cholesterol; HOMA-IR, homeostasis model assessment of insulin resistance; LDL-C, high-density-lipoprotein cholesterol; TC: HDL-C, ratio of total to high-density-lipoprotein cholesterol, TG: HDL-C, ratio of triglycerides to high-density-lipoprotein cholesterol; WC, waist circumference; ^1^ Models adjusted for age at recruitment and ethnicity; education, household income, physical activity, parity and their changes; and booking BMI, pregnancy diet quality, and gestational-weight-gain category; ^2^ n = 93 “large decrease”, n = 84 “small decrease”, n = 155 “stable”, n = 89 “small increase”, n = 62 “large increase”; ^3^ n = 109 “large decrease”, n = 98 “small decrease”, n = 175 “stable”, n = 106 “small increase”, n = 78 “large increase”; ^4^ Models adjusted for age at recruitment and ethnicity; education, household income, physical activity, parity and their changes; and booking BMI, pregnancy diet quality, weight changes at year 8, ^5^ GDM, and ^6^ hypertensive disorders of pregnancy.

**Table 3:** Associations between diet quality at pregnancy or at 6 years postpregnancy and anthropometry and cardiometabolic markers at 6–8 years postpregnancy in women of the GUSTO cohort.

	Q1	Q2		Q3		Q4	
		β (95% CI)	*p*	β (95% CI)	*p*	β (95% CI)	*p*
Diet quality at pregnancy							
Anthropometry ^1^							
BMI, kg/m^2^	Reference	−0.33 (−0.96, 0.30)	0.300	−0.21 (−0.84, 0.42)	0.512	−0.62 (−1.25, 0.01)	0.056
Skinfolds ^2^, mm	Reference	0.40 (−3.90, 4.70)	0.856	−1.59 (−5.98, 2.80)	0.477	−4.75 (−9.13, −0.37)	0.033
WC ^3^, cm	Reference	−0.44 (−2.14, 1.26)	0.610	0.28 (−1.42, 1.97)	0.748	−1.97 (−3.70, −0.25)	0.025
Lipid profile ^4^							
Total cholesterol, mmol/L	Reference	−0.05 (−0.26, 0.15)	0.616	−0.04 (−0.25, 0.17)	0.698	−0.14 (−0.35, 0.07)	0.191
Triglycerides, mmol/L	Reference	−0.08 (−0.20, 0.04)	0.187	−0.11 (−0.23, 0.01)	0.072	−0.21 (−0.33, −0.09)	0.001
LDL-C, mmol/L	Reference	−0.02 (−0.19, 0.16)	0.863	0.02 (−0.16, 0.20)	0.856	−0.08 (−0.26, 0.10)	0.377
HDL-C, mmol/L	Reference	−0.001 (−0.07, 0.06)	0.965	−0.01 (−0.08, 0.06)	0.796	0.04 (−0.03, 0.10)	0.309
TC: HDL-C	Reference	−0.07 (−0.26, 0.12)	0.463	−0.05 (−0.24, 0.15)	0.646	−0.22 (−0.42, −0.02)	0.030
TG: HDL-C	Reference	−0.08 (−0.19, 0.04)	0.199	−0.10 (−0.22, 0.02)	0.096	−0.21 (−0.33, −0.09)	0.001
Glycemia ^5^							
Fasting glucose, mmol/L	Reference	0.09 (−0.17, 0.35)	0.502	−0.18 (−0.45, 0.09)	0.181	−0.24 (−0.52, 0.03)	0.083
HOMA-IR	Reference	0.11 (−0.30, 0.52)	0.597	−0.06 (−0.48, 0.36)	0.782	−0.35 (−0.78, −0.08)	0.017
Blood pressure ^6^							
Systolic, mmHg	Reference	1.94 (−1.20, 5.08)	0.225	−0.30 (−3.47, 2.86)	0.850	0.92 (−2.30, 4.15)	0.575
Diastolic, mmHg	Reference	1.56 (−0.73, 3.84)	0.181	−0.61 (−2.92, 1.69)	0.603	0.18 (−2.17, 2.53)	0.879
Diet quality at 6 years postpregnancy							
Anthropometry ^7^							
BMI, kg/m^2^	Reference	0.19 (−0.34, 0.72)	0.481	0.17 (−0.35, 0.71)	0.512	−0.35 (−0.90, 0.20)	0.208
Skinfold ^2^, mm	Reference	1.59 (−4.16, 7.33)	0.587	−3.37 (−9.11, 2.36)	0.249	−3.80 (−9.58, 1.97)	0.196
WC ^3^, cm	Reference	−1.03 (−2.71, 0.66)	0.231	−0.13 (−1.82, 1.57)	0.884	−1.48 (−3.20, 0.25)	0.093
Lipid profile ^8^							
Total cholesterol, mmol/L	Reference	0.03 (−0.17, 0.24)	0.738	−0.06 (−0.27, 0.15)	0.551	−0.15 (−0.36, 0.05)	0.144
Triglycerides, mmol/L	Reference	−0.10 (−0.22, 0.02)	0.090	−0.07 (−0.19, 0.05)	0.242	−0.15 (−0.28, −0.03)	0.012
LDL-C, mmol/L	Reference	0.02 (−0.15, 0.20)	0.788	−0.05 (−0.23, 0.13)	0.556	−0.11 (−0.29, 0.07)	0.226
HDL-C, mmol/L	Reference	0.06 (−0.01, 0.12)	0.085	0.02 (−0.04, 0.09)	0.503	0.03 (−0.04, 0.09)	0.460
TC: HDL-C	Reference	−0.16 (−0.35, 0.03)	0.091	−0.16 (−0.35, 0.04)	0.113	−0.23 (−0.43, −0.04)	0.018
TG: HDL-C	Reference	−0.09 (−0.22, 0.02)	0.095	−0.11 (−0.23, 0.01)	0.084	−0.17 (−0.30, −0.05)	0.005
Glycemia ^9^							
Fasting glucose, mmol/L	Reference	−0.06 (−0.17, 0.06)	0.340	−0.03 (−0.16, 0.09)	0.603	−0.04 (−0.17, 0.08)	0.479
HOMA-IR	Reference	−0.20 (−0.49, 0.09)	0.169	−0.18 (−0.48, 0.02)	0.074	−0.24 (−0.65, −0.08)	0.039
Blood pressure ^10^							
Systolic, mmHg	Reference	2.47 (−0.55, 5.50)	0.108	0.80 (−2.23, 3.84)	0.604	0.52 (−2.58, 3.62)	0.742
Diastolic, mmHg	Reference	1.76 (−0.50, 4.03)	0.126	0.55 (−1.73, 2.82)	0.637	0.18 (−2.14, 2.50)	0.877

BMI, body mass index; GUSTO, Growing Up in Singapore Towards healthy Outcomes; HDL-C, high-density-lipoprotein cholesterol; HOMA-IR, homeostasis model assessment of insulin resistance; LDL-C, high-density-lipoprotein cholesterol; TC: HDL-C, ratio of total to high-density-lipoprotein cholesterol, TG: HDL-C, ratio of triglycerides to high-density-lipoprotein cholesterol; WC, waist circumference; ^1^ Models adjusted for age at recruitment and ethnicity; and education, household income, parity, physical activity during pregnancy; and booking BMI, diet quality at year 6, and gestational-weight-gain category; ^2^ n = 126 Q1, n = 122 Q2, n = 123 Q3, n = 112 Q4; ^3^ n = 146 Q1, n = 144 Q2, n = 141 Q3, n = 135 Q4; ^4^ Models adjusted for age at recruitment and ethnicity; and education, household income, parity, physical activity during pregnancy, booking BMI, diet quality at year 6, weight changes at year 8, and ^5^ GDM or ^6^ hypertensive disorders of pregnancy; ^7^ Models adjusted for age at recruitment and ethnicity; education, household income, and parity at years 4–5; and physical activity at year 6, diet quality at pregnancy, booking BMI, and gestational-weight-gain category; ^8^ Models adjusted for age at recruitment and ethnicity; education, household income, and parity at years 4–5; and physical activity at year 6, diet quality at pregnancy, booking BMI, weight changes at year 8, and ^9^ GDM or ^10^ hypertensive disorders of pregnancy.

## Data Availability

Data described in the manuscript, code book, and analytic code will be made available upon request, pending approval by the lead investigators of the GUSTO study.

## Supplementary Materials

The following supporting information can be downloaded at: https://www.mdpi.com/article/10.3390/nu15081870/s1. Table S1: Comparison of HEI-SGP scoring criteria between pregnancy and 6 years postpregnancy; Table S2: Associations between change in diet quality from pregnancy to 6 years postpregnancy and anthropometry and cardiometabolic markers at 6–8 years postpregnancy in women of the GUSTO cohort, stratified by parity; Table S3: Anthropometry and cardiometabolic markers at 6–8 years postpregnancy according to 6 groups of change in diet quality from pregnancy to 6 years postpregnancy in women of the GUSTO cohort; Figure S1: The association between change in diet quality, successively excluding individual HEI-SGP components, and total and LDL cholesterol in GUSTO women; Figure S2: The associations between change in diet quality, successively excluding individual HEI-SGP components, and triglycerides, triglycerides: HDL-C ratio, and HOMA-IR in GUSTO women.
