# Supplementary material for: Changes in Diet Quality from Pregnancy to 6 Years Postpregnancy and Associations with Cardiometabolic Risk Markers
Source: Nutrients. 2023 Apr 13;15(8):1870. doi: 10.3390/nu15081870 (PMC10145322; doi:10.3390/nu15081870)
Supplement: Supplementary file 1 [file nutrients-15-01870-s001.zip › nutrients-2301997-supplementary.pdf]

Supplementary Material

Changes in Diet Quality from Pregnancy to 6 Years Postpregnancy and Associations with Cardiometabolic Risk Markers

Jun S. Lai [1,*], Marjorelee T. Colega [1], Keith M. Godfrey [2], Kok Hian Tan [3], Fabian Yap [4,5], Yap Seng Chong [1,6], Yung Seng Lee [7], Johan G. Eriksson [1,6,8], Shiao-Yng Chan [1,6] and Mary F. F. Chong [1,9]

1 Singapore Institute for Clinical Sciences, Agency for Science Technology and Research, Singapore 117609, Singapore; marjorelee_colega@sics.a-star.edu.sg (M.T.C.); obgcys@nus.edu.sg (Y.S.C.); obgjge@nus.edu.sg (J.G.E.); obgchan@nus.edu.sg (S.-Y.C.); mary_chong@nus.edu.sg (M.F.F.C.)
2 MRC Lifecourse Epidemiology Centre & NIHR Southampton Biomedical Research Centre, University of Southampton & University Hospital Southampton NHS Foundation Trust, Southampton SO16 6YD, UK; kmg@mrc.soton.ac.uk
3 Department of Maternal Fetal Medicine, KK Women's and Children's Hospital, Singapore 229899, Singapore; tan.kok.hian@singhealth.com.sg
4 Duke-NUS Medical School, Singapore 169857, Singapore; fabian.yap.k.p@singhealth.com.sg
5 Department of Paediatric Endocrinology, KK Women's and Children's Hospital, Singapore 229899, Singapore
6 Department of Obstetrics & Gynaecology and Human Potential Translational Research Programme, Yong Loo Lin School of Medicine, National University of Singapore and National University Health System, Singapore 119228, Singapore
7 Department of Paediatrics, Yong Loo Lin School of Medicine, National University of Singapore and National University Health System, Singapore 119228, Singapore; paeleeys@nus.edu.sg
8 Finland and Folkhälsan Research Center, University of Helsinki, Helsinki 00014, Finland
9 Saw Swee Hock School of Public Health, National University of Singapore and National University Health System, Singapore 117549, Singapore
* Correspondence: lai_jun_shi@sics.a-star.edu.sg

Table S1. Comparison of HEI-SGP scoring criteria between pregnancy and 6 years post-pregnancy.

HEI-SGP component	Score	Scoring criteria	
		Pregnancy[a]	Year-6[b]
Total rice and alternatives	0 – 10	≥ 2.60 serves / 1000 kcal	≥ 2.50 serves / 1000 kcal
Whole grains	0 – 10	≥ 1.30 serves / 1000 kcal	≥ 1.25 serves / 1000 kcal
Total fruit	0 – 5	≥ 0.87 serves / 1000 kcal	≥ 1 serve / 1000 kcal
Whole fruit	0 – 5	≥ 0.43 serves / 1000 kcal	≥ 0.5 serves / 1000 kcal
Total vegetables	0 – 5	≥ 1.30 serves / 1000 kcal	≥ 1 serve / 1000 kcal
Dark green and orange vegetables	0 – 5	≥ 0.21 serves / 1000 kcal	≥ 0.21 serves / 1000 kcal
Total protein foods	0 – 10	≥ 1.08 serves / 1000 kcal	≥ 1 serve / 1000 kcal
Dairy	0 – 10	≥ 0.43 serves / 1000 kcal	≥ 0.25 serves / 1000 kcal
Total fat	0 – 10	≤ 30% energy	≤ 30% energy
Saturated fat	0 – 10	≤ 10% energy	≤ 10% energy
Use of antenatal supplements	0 – 5 – 10	Consume supplements containing iron, folate, and calcium	Not applicable

HEI-SGP, Healthy Eating Index for Pregnant women in Singapore

[a]Based on average energy requirement of 2300kcal

[b]Based on average energy requirement of 2000kcal

Table S2. Associations of change in diet quality from pregnancy to 6 years post-pregnancy with anthropometry and cardio-metabolic markers at 6-8 years post-pregnancy in women of the GUSTO cohort, stratified by parity.

	**Large decrease**	**Small decrease**	**Stable**	**Small increase**	**Large increase**	
	β (95% CI)	β (95% CI)		β (95% CI)	β (95% CI)	P-interaction
BMI[a], kg/m^2						
0	-0.22 (-1.25, 0.81)	0.29 (-0.71, 1.29)	Reference	0.33 (-0.55, 1.21)	-0.50 (-1.57, 0.06)	0.152
≥1	-0.13 (-0.95, 0.69)	0.12 (-0.62, 0.86)		0.14 (-0.60, 0.88)	-0.84 (-1.66, -0.01)	
Skinfolds[a], mm						
0	-1.32 (-9.41, 6.77)	-0.49 (-7.97, 6.99)	Reference	1.67 (-5.35, 8.69)	0.20 (-8.45, 8.84)	0.590
≥1	2.45 (-4.43, 9.32)	4.64 (-1.59, 10.87)		4.31 (-1.85, 10.46)	-1.19 (-8.23, 5.86)	
WC[a], cm						
0	-0.38 (-3.54, 2.78)	0.37 (-2.68, 3.43)	Reference	0.40 (-2.30, 3.09)	0.51 (-2.76, 3.78)	0.512
≥1	0.94 (-1.72, 3.60)	2.35 (-0.05, 4.64)		1.23 (-1.17, 3.63)	-2.04 (-4.76, 0.68)	
Cholesterol[b], mmol/L						
0	0.05 (-0.35, 0.45)	0.07 (-0.36, 0.50)	Reference	-0.26 (-0.61, 0.09)	-0.45 (-0.91, 0.003)	0.023
≥1	0.50 (0.19, 0.81)*	-0.11 (-0.41, 0.18)		0.22 (-0.06, 0.51)	0.24 (-0.07, 0.54)	
Triglycerides[b], mmol/L						
0	-0.08 (-0.30, 0.14)	0.02 (-0.22, 0.25)	Reference	-0.12 (-0.32, 0.07)	-0.25 (-0.50, -0.01)	0.778
≥1	0.06 (-0.13, 0.25)	0.01 (-0.17, 0.19)		-0.11 (-0.28, 0.07)	-0.11 (-0.30, 0.08)	
LDL-C[b], mmol/L						
0	0.04 (-031, 0.38)	0.11 (-0.27, 0.48)	Reference	-0.19 (-0.50, 0.11)	-0.24 (-0.63, 0.15)	0.010

≥1	0.41 (0.14, 0.68)*	-0.12 (-0.37, 0.13)		0.18 (-0.06, 0.43)	0.18 (-0.07, 0.44)	
HDL-C[b], mmol/L						
0	0.04 (-0.10, 0.18)	-0.03 (-0.17, 0.12)	Reference	-0.01 (-0.13, 0.11)	-0.04 (-0.20, 0.11)	0.428
≥1	0.06 (-0.04, 0.15)	0.005 (-0.09, 0.09)		0.08 (-0.01, 0.16)	0.08 (-0.01, 0.18)	
TC: HDL-C[b]						
0	-0.19 (-0.54, 0.15)	0.16 (-0.21, 0.52)	Reference	-0.17 (-0.47, 0.13)	-0.21 (-0.59, 0.17)	0.150
≥1	0.25 (-0.04, 0.55)	-0.14 (-0.41, 0.14)		-0.11 (-0.38, 0.15)	-0.12 (-0.41, 0.17)	
TG: HDL-C[b]						
0	-0.06 (-0.27, 0.15)	0.05 (-0.17, 0.27)	Reference	-0.08 (-0.26, 0.11)	-0.23 (-0.46, 0.01)	0.126
≥1	0.03 (-0.16, 0.22)	-0.004 (-0.19, 0.18)		-0.18 (-0.35, 0.001)	-0.17 (-0.36, 0.02)	
Fasting glucose[c], mmol/L						
0	-0.60 (-1.22, 0.03)	-0.37 (-0.89, 0.15)	Reference	-0.30 (-1.03, 0.43)	-0.40 (-1.02, 0.21)	0.627
≥1	0.11 (-0.09, 0.31)	0.20 (-0.14, 0.53)		0.14 (-0.40, 0.67)	-0.17 (-0.41, 0.08)	
HOMA-IR[c]						
0	0.57 (-0.13, 1.28)	0.55 (-0.06, 1.15)	Reference	-0.22 (-0.81, 0.37)	-0.47 (-1.22, 0.27)	0.100
≥1	1.01 (0.35, 1.67)	-0.07 (-0.62, 0.49)		-0.40 (-0.94, 0.13)	-0.59 (-1.16, -0.03)	
Systolic BP[d], mmHg						
0	-3.84 (8.46, 0.77)	-3.36 (-8.29, 1.57)	Reference	-3.37 (-7.50, 0.76)	0.32 (-4.98, 5.62)	0.513
≥1	0.03 (-5.26, 5.31)	4.29 (-0.64, 9.22)		-1.86 (-6.67, 2.94)	0.17 (5.04, 5.38)	
Diastolic BP[d], mmHg						
0	-2.98 (-6.91, 0.95)	-3.19 (-7.37, 0.99)	Reference	-3.06 (-7.06, 0.05)	-0.82 (-5.33, 3.68)	0.313

≥1	-1.31 (-5.01, 2.39)	1.81 (-1.65, 5.26)		-3.13 (-6.49, 0.24)	-0.03 (-3.68, 3.61)

BMI, body mass index; BP, blood pressure; HDL-C, high-density-lipoprotein cholesterol; HOMA-IR, homeostasis model assessment of insulin resistance; LDL-C, high-density-lipoprotein cholesterol; TC: HDL-C, ratio of total to high-density-lipoprotein cholesterol, TG: HDL-C, ratio of triglycerides to high-density-lipoprotein cholesterol; WC, waist circumference

Models adjusted for age at recruitment, ethnicity; education, household income, physical activity and their changes; booking BMI, pregnancy diet quality; [a]gestational weight gain category, [b]weight changes at Year-8 and [c]GDM or [d]hypertensive disorders of pregnancy

*P<0.05

Table S3. Anthropometry and cardio-metabolic markers at 6-8 years post-pregnancy according to 6 groups of change in diet quality from pregnancy to 6 years post-pregnancy in women of the GUSTO cohort.

	**Maintained low**	**Large decrease**	**Small decrease**	**Small increase**	**Large increase**	**Maintained high**
	Mean ± SD	Mean ± SD	Mean ± SD	Mean ± SD	Mean ± SD	Mean ± SD
Anthropometry	n=95	n=102	n=92	n=111	n=90	n=79
BMI, kg/m^2	25.5 ± 5.5	24.1 ± 4.5	24.3 ± 4.6	26.9 ± 6.9	25.0 ± 5.1	23.8 ± 4.6
Skinfolds[a], mm	73.4 ± 21.4	68.8 ± 23.5	71.4 ± 23.2	80.1 ± 25.5	71.2 ± 23.9	68.3 ± 20.6
Waist circumference[b], cm	84.8 ± 11.8	83.5 ± 10.5	83.9 ± 10.3	87.8 ± 12.0	84.2 ± 10.7	82.5 ± 9.7
Lipid profile	n=81	n=92	n=76	n=97	n=80	n=64
Total cholesterol, mmol/L	4.85 ± 0.79	4.99 ± 0.99	4.64 ± 0.81	4.87 ± 0.82	4.80 ± 0.73	4.63 ± 0.67
Triglycerides, mmol/L	1.18 ± 0.70	0.95 ± 0.39	0.98 ± 0.51	1.03 ± 0.50	0.99 ± 0.44	0.93 ± 0.41
LDL-C, mmol/L	2.97 ± 0.64	3.12 ± 0.87	2.86 ± 0.56	3.03 ± 0.69	2.98 ± 0.66	2.82 ± 0.66
HDL-C, mmol/L	1.34 ± 0.32	1.34 ± 0.27	1.37 ± 0.28	1.37 ± 0.28	1.37 ± 0.27	1.44 ± 0.35
TC: HDL-C	3.76 ± 0.88	3.61 ± 0.99	3.56 ± 0.75	3.66 ± 0.80	3.62 ± 0.83	3.47 ± 0.76
TG: HDL-C	0.96 ± 0.68	0.75 ± 0.45	0.78 ± 0.53	0.81 ± 0.52	0.77 ± 0.43	0.72 ± 0.40
Glycemia	n=78	n=89	n=74	n=95	n=77	n=62
Fasting glucose, mmol/L	5.13 ± 1.68	4.91 ± 0.49	4.97 ± 0.97	5.19 ± 1.45	4.87 ± 0.52	4.87 ± 0.47
HOMA-IR	8.60 ± 5.75	7.49 ± 5.80	7.69 ± 5.32	8.27 ± 5.96	7.71 ± 6.78	7.32 ± 5.89
Blood pressure	n=85	n=101	n=87	n=113	n=86	n=71

Systolic, mmHg	114 ± 13	110 ± 12	114 ± 18	112 ± 13	112 ± 14	109 ± 12
Diastolic, mmHg	69 ± 10	66 ± 9	68 ± 12	67 ± 9	68 ± 10	65 ± 9

BMI, body mass index; GUSTO, Growing Up in Singapore Towards healthy Outcomes; HDL-C, high-density-lipoprotein cholesterol; HOMA-IR, homeostasis model assessment of insulin resistance; LDL-C, high-density-lipoprotein cholesterol; TC: HDL-C, ratio of total to high-density-lipoprotein cholesterol, TG: HDL-C, ratio of triglycerides to high-density-lipoprotein cholesterol; WC, waist circumference

[a] n=81 ‘maintained low’, n=85 ‘large decrease’, n=78 ‘small decrease, n=93 ‘small increase’, n=70 ‘large increase’, n=73 ‘maintained high’

[b] n=94 ‘maintained low’, n=102 ‘large decrease’, n=91 ‘small decrease’, n=110 ‘small increase’, n=86 ‘large increase’, n=79 ‘maintained high’

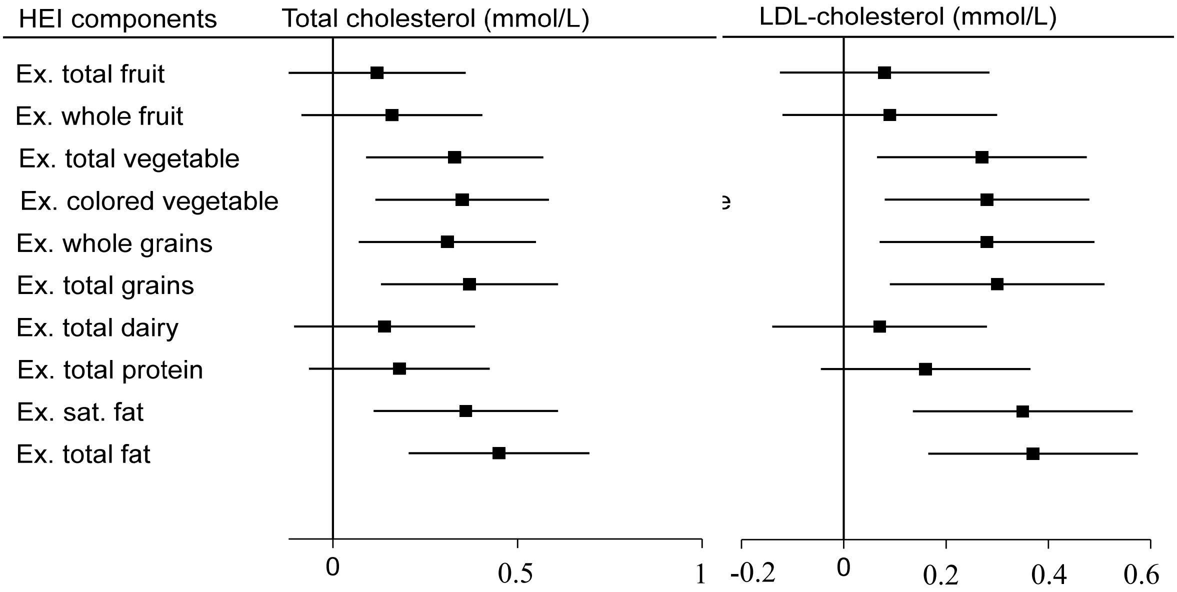

Figure S1. The association of change in diet quality, successively excluding individual HEI-SGP components, with total and LDL-cholesterol in GUSTO women. Black squares denote linear regression coefficients and horizontal lines denote 95% CIs. The models were adjusted for age at recruitment, ethnicity; education, household income, parity, physical activity and their changes; booking BMI, pregnancy HEI, weight retention at Year-8. HEI, Healthy Eating Index; GUSTO, Growing Up in Singapore Towards healthy Outcomes; LDL, low density lipoprotein.

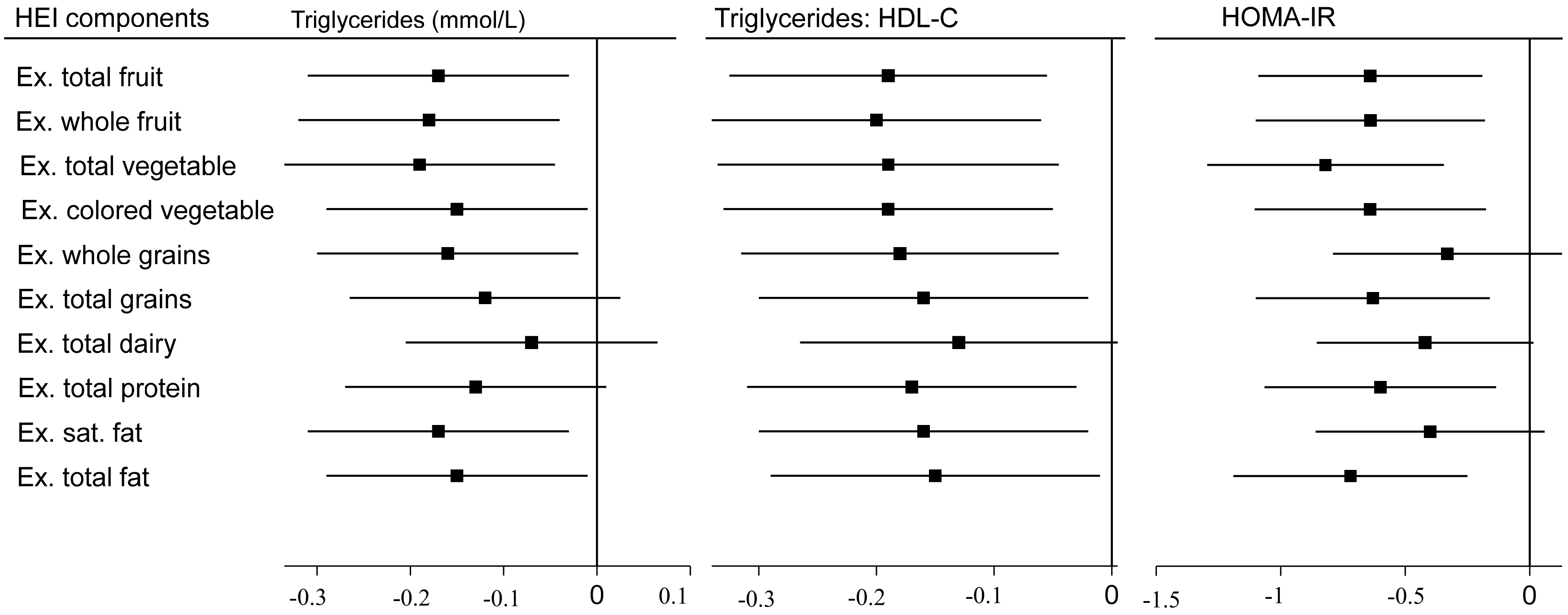

Figure S2. The associations of change in diet quality, successively excluding individual HEI-SGP components, with triglycerides, triglycerides: HDL-C ratio and HOMA-IR in GUSTO women. Black squares denote linear regression coefficients and horizontal lines denote 95% CIs. The models were adjusted for age at recruitment, ethnicity; education, household income, parity, physical activity and their changes; booking BMI, pregnancy HEI, weight retention at Year-8, and additionally for GDM for analysis with HOMA-IR. HDL-C, high density lipoprotein cholesterol; HEI, Healthy Eating Index; HOMA-IR, homeostasis model assessment of insulin resistance; GUSTO, Growing Up in Singapore Towards healthy Outcomes.
